# When Is the Right Time to End Family Therapy for Anorexia Nervosa (FT‐AN)?: A Qualitative Study of Young People's Experiences

**DOI:** 10.1002/erv.70025

**Published:** 2025-09-03

**Authors:** Kate de la Poer Beresford, Alys Cawson, Julian Baudinet, Ivan Eisler, Anna Konstantellou, Mima Simic, Ulrike Schmidt

**Affiliations:** ^1^ Maudsley Centre for Child and Adolescent Eating Disorders (MCCAED) South London and Maudsley NHS Foundation Trust London UK; ^2^ Centre for Research in Eating and Weight Disorders (CREW) Institute of Psychiatry Psychology and Neuroscience (IoPPN) King's College London London UK; ^3^ Adult Eating Disorders Service South London and Maudsley NHS Foundation Trust London UK

**Keywords:** adolescent, anorexia nervosa, child, eating disorders, family based treatment (FBT), family therapy for anorexia nervosa (FT‐AN), Maudsley family therapy

## Abstract

**Objective:**

Family therapy for anorexia nervosa (FT‐AN) is the first‐line recommended treatment for young people with anorexia nervosa. There is variability in treatment length across studies and evidence suggests treatment length and outcome are not necessarily linearly related. This makes it difficult to identify the optimum length of treatment in clinical practice. This study aimed to explore young people's perspectives on the timing of discharge and how this relates to recovery.

**Method:**

Twenty three young people (age 12–18) diagnosed with anorexia (or atypical anorexia) nervosa participated. All had completed FT‐AN with or without adjunctive multi‐family therapy. Semi‐structured individual qualitative interviews were conducted. Recordings were transcribed verbatim and analysed using reflexive thematic analysis.

**Results:**

Four inter‐connected themes were generated; (1) who decides?, (2) knowing what's coming, (3) things that need to be in place, (4) discharge is a necessary step towards recovery.

**Discussion:**

Young people said that remaining in treatment for longer than necessary may impede recovery. Establishing clear expectations about discharge and recovery, helping young people to commit to ongoing behaviour change, and building their support network were all described as important components in helping them to feel confident about discharge and to take ownership of continuing the recovery journey post‐discharge.

## Key Points

- Findings suggest ending family therapy for anorexia nervosa (FT‐AN) is not about attending a specific number of sessions or reaching a certain weight; rather, it is about supporting young people and their families to feel confident in their ability to manage without treatment.
- Ongoing commitment to change post‐discharge is paramount, and encouraging young people and families to take ‘safe risks’ seems to be an important part of ending and taking ownership of recovery.
- Current data suggests holding young people in treatment longer than necessary may inadvertently signal that change is too risky and that the multi‐disciplinary team do not think they are able to cope without ongoing support.

## 1 Introduction

Family therapy for anorexia nervosa is the first line recommended treatment for child and adolescent eating disorders in most international guidelines (Hilbert et al. 2017). Several different versions exist, including Maudsley family therapy for anorexia nervosa (FT‐AN; Eisler, Simic, Blessitt, et al. 2016), family based treatment (FBT; Lock and Le Grange 2012), parent‐focused therapy (PFT; Le Grange et al. 2016), and multi‐family therapy (MFT; Simic et al. 2021). What unites these interventions is the emphasis on the family as a resource in treatment and recovery, the phased nature of treatment, the prioritisation of physical health recovery in the early phases and a shift to a broader focus on individual development and family life‐cycle issues in later stages of the treatment (Gorrell et al. 2023).

Despite these similarities there have been quite large differences regarding the number of sessions and treatment length reported in the literature. In published randomised controlled trials (RCTs) the number of sessions have ranged from 10 (Lock et al. 2005; Russell et al. 1987) to 24 (Lock et al. 2010) over a duration of six (Le Grange et al. 2016; Lock et al. 2005, 2015; Russell et al. 1987), nine (Agras et al. 2014), 12 (Eisler, Simic, Hodsoll, et al. 2016; Lock et al. 2005, 2010; Madden et al. 2015) and 18 months (Godart et al. 2012; Robin et al. 1999). In the FBT manual it is said that treatment may be as few as 10 sessions over 6 months, with longer treatment indicated for those with more obsessive compulsive features (Lock and Le Grange 2012, 22). Nevertheless, it is also reported in the FBT manual that phase 1, focused on weight restoration, can range from as few as two to three sessions to 10 or more (Lock and Le Grange 2012, 32). In the FT‐AN manual, it is said that treatment is usually six to nine months with no session number prescribed (Eisler, Simic, Blessitt, et al. 2016, 41).

Given the variability in duration of treatment in both published trials and treatment manuals, it brings into question what the optimal treatment length is and how to make decisions about discharge in an evidence‐informed way. The decision around when to end treatment can be difficult given a lack of consensus in the field regarding how recovery is defined (Bardone‐Cone et al. 2018). There is general consensus that recovery includes physical, psychological and behavioural elements; however, the exact parameters are still debated. This poses challenges in clinical practice, with many clinicians and families faced with the question: *when is enough enough*?

In many medical interventions, there can be an expectation that treatment ends when there is full remission of symptoms. However, this may not fully translate to mental health interventions for two reasons. Firstly, there is evidence from both the field of psychotherapy in general (Robinson et al. 2020) and eating disorders more specifically (e.g. Bell et al. 2017) that there is not a linear relationship between duration of treatment and outcome. Most changes in psychological therapies occur early in treatment, with only limited additional gains observed after a certain number of sessions, although this varies by the type of treatment and the condition being treated. Linked to this are findings that, for most people, brief treatments are as effective as the same treatment delivered over a longer period (Allen et al. 2025; Hansen et al. 2002; Keegan et al. 2022; Lock et al. 2005). Secondly, the manner in which therapy is brought to a conclusion is itself an important part of treatment and is likely to have an ongoing impact on the continuing process of recovery post‐treatment. In clinical practice, therefore, the length of treatment is often more varied, and it is not unusual to end treatment when there is significant improvement and a recognition that some further progress may still be needed post‐discharge (Bear et al. 2022). While ending treatment too early, before sufficient progress has been made, can undoubtedly sometimes increase the risk of relapse, there is also evidence that prolonged treatment may often fail to produce any further benefits and, in some cases, actually reduce the chance of recovery (Saxon et al. 2017).

This is important as relapse rates in eating disorders have been reported to be relatively high. In a recent systematic review on what happens post‐discharge from eating disorder treatment, relapse rates were reported to range from 9% to 52%, with the rate increasing with longer duration of follow‐up (Khalsa et al. 2017). The authors also reported that the first year post‐discharge was a higher risk time for relapse, which might indicate that terminating treatment too soon may have deleterious effects. Nevertheless, in a recent naturalistic follow‐up study of a UK‐based specialist child and adolescent eating disorder service (cf. Simic et al. 2022 for end‐of‐treatment outcomes), rates of self‐reported eating disorder diagnoses were relatively low (6.7%) at a mean follow‐up period of 7 years (Stewart et al. 2022) and overall only 10% reported that eating disorder difficulties in general continued to have a significant impact on their lives.

To better understand the process of ending therapy, the current study aimed to qualitatively evaluate young people's perspectives on the timing of discharge from FT‐AN and how this relates to self‐reported recovery.

## 2 Method

The Stanmore Research Ethics Committee London (IRAS: 234354; REC: 20/LO/0839) provided ethical approval for this study. All participants provided written informed consent or assent.

### 2.1 Sample

This study used a combined sample from two previous qualitative studies (Baudinet, Eisler, et al. 2023; Baudinet et al. 2024). These studies examined how young people perceived change to occur in FT‐AN with or without the addition of multi‐family therapy for anorexia nervosa (MFT‐AN; Simic et al. 2021) as part of their outpatient treatment at the Maudsley Centre for Child and Adolescent Eating Disorders (MCCAED). In addition to their understanding of change processes during treatment (the focus of previous publications), all participants were also asked during the interviews about their experience of ending treatment, their views on recovery and what it means to them.

Young people (12–18 years) were eligible for this study if they (a) had a diagnosis of anorexia nervosa or atypical anorexia nervosa, (b) received FT‐AN with or without MFT‐AN as part of their outpatient treatment, and (c) completed treatment within the last 2 years. MCCAED is a specialist child and adolescent eating disorder services in London, UK, covering the area of southeast London and a population of approximately 2 million people.

Participants were identified by the clinical team. After being approached by their clinician, if the young person was interested, the research group received consent to contact. Up to three attempts at contact were made per person.

### 2.2 Recruitment

All young people who completed FT‐AN with or without MFT‐AN at MCCAED were approached to participate, regardless of treatment outcome, length or engagement. The initial study recruited participants between June and December 2021 (Baudinet, Eisler, et al. 2023); the latter between July 2022 and February 2023 (Baudinet et al. 2024).

### 2.3 Data Collection

Qualitative interviews were conducted with all participants individually for those who provided consent (≥ 16 years) or assent (< 16 years). All interviews were conducted via video‐call by authors J.B (male, clinical psychologist, DClinPsych and PhD), AK (female, assistant psychologist, PhD), or two other assistant psychologists in MCCAED. Each interview lasted approximately 60 min with only the interviewer and the participant present. Interviews were recorded and then transcribed verbatim. All interviews followed the same topic guide and pilot interviews were conducted to ensure uniformity between interviewers. Transcriptions were not returned to participants for review.

For any cases where J.B. was involved in delivering treatment, other research team members conducted the interviews. To mitigate potential bias, all interview transcripts were anonymised during transcription, which was performed by an external service. The anonymised transcripts were then used for analysis.

### 2.4 Diagnostic Assessment and Screening

Before their initial assessment at the clinic, all participants completed the Development and Well‐being Assessment (DAWBA) (Goodman et al. 2000). The DAWBA is a structured diagnostic tool that generates DSM‐5 (American Psychiatric Association 2013) and ICD‐10 (World Health Organization 2004) criteria for individuals up to and including age 17. Clinical assessment at MCCAED confirmed the eating disorder diagnoses.

### 2.5 Treatment Description

Participants in this study received manualised outpatient FT‐AN (Eisler, Simic, Blessitt, et al. 2016), with or without MFT‐AN (Simic et al. 2021), as part of routine care provided at the NHS outpatient clinic. FT‐AN consists of four treatment phases: initial engagement (phase 1), symptom management (phase 2), addressing broader adolescent needs and life cycle changes (phase 3) and ending therapy with a focus on relapse prevention (phase 4). Treatment begins with weekly sessions and transitions to less frequent meetings as progress is made. Early phases prioritise supporting families in managing eating disorder symptoms, facilitating weight restoration (if necessary), and building distress tolerance skills. The later phases are tailored to the specific needs of each family and may address issues such as independent eating, school and peer relationships, and managing uncertainty. The frequency and number of FT‐AN sessions are adjusted based on the family's clinical needs. MFT‐AN draws upon the same theoretical framework as FT‐AN but extends the model by offering an intensive group‐based format as an adjunct to FT‐AN (cf. Baudinet, Eisler, et al. 2021; Baudinet and Eisler 2024b; Zinser et al. 2022 for reviews). MFT‐AN increases support for the whole family and reduces perceived isolation (Dawson et al. 2018), a common experience for people with anorexia nervosa and their family members. Participants in MFT‐AN continued to receive concurrent FT‐AN sessions.

### 2.6 Analysis Plan and Reflexivity Statement

The data generated were analysed by K.B. (MPsych (Clin), white Australian female) and A.C. (MD, white British female), under the supervision of J.B., using reflexive thematic analysis (Braun and Clarke 2020). The analysis was conducted within a critical realist framework, which acknowledges that experiences and meanings are subjective and shaped by socio‐cultural contexts. K.B. had several years of clinical experience delivering FT‐AN and MFT‐AN in the UK and Australia. A.C. had several years of experience in child and adolescent psychiatric settings, and had been delivering FT‐AN for 2 years. In supervising the data, J.B. brought extensive knowledge of both FT‐AN and MFT‐AN, with many years of clinical experience, research and teaching on the models.

The analysis adhered to Braun and Clarke's (2006) six‐phase framework for thematic analysis. This process involved familiarisation with the data, memos, independent coding, and the identification of preliminary themes by each analysing author. Through iterative discussions in two one‐hour meetings, themes were refined, and a final consensus was reached. Initial themes and codes aligned well, and consensus on final themes was reached through discussion between A.C. and K.B., supervised by J.B.

A flexible, deductive approach was employed (Fletcher 2017) drawing on established systemic theories and literature relevant to FT‐AN. The analysis did not rely on the concept of data saturation, recognising the inherently subjective nature of theme generation (Braun and Clarke 2021). No software was used for the analysis.

## 3 Results

Of the 70 eligible families identified during the recruitment periods, the clinical team deemed three to be inappropriate to contact due to ongoing distress or risk issues. Contact was attempted with the remaining 67. Nine of these were unable to be reached after a maximum of three attempts by a clinical staff member. Of the remaining 58, 17/58 (29.3%) actively declined, 18/58 (31.0%) passively declined (provided initial consent or requested further information but were unreachable or unavailable to complete an interview thereafter), and 23/58 (39.7%) consented to participate and completed an interview. Reasons for non‐participation included not having time to participate, not being interested in the study, or not wanting to discuss or revisit their treatment journey.

See Table 1 for demographic and treatment details for the included sample. All (23/23, 100%) were living at home at the time of study participation. Seventeen (73.9%) were living with two parents at home, and six (26.1%) had parents who were separated. None identified as living with a disability.

**TABLE 1:** Demographic and treatment characteristics.

	Mean	(SD, range)
Age		
Assessment	14.81	1.37, 12 – 18
Interview	16.26	1.49, 13 – 19
Missing (*n* = 0)	—	—
%mBMI		
Assessment	85.45	8.66, 70.00 – 105.39
Discharge	94.77	7.08, 81.00 – 109.41
Missing (*n* = 0)	—	—
Treatment characteristics		
Months	8.87	4.87, 3 – 22
FT‐AN sessions	17.70	9.19, 5 – 41
MFT days^a^	5.50	1.31, 4 – 8
Missing (*n* = 0)	—	—

	*N*	%
Gender		
Female	21	91.3
Male	1	4.3
Gender fluid	1	4.3
Missing (*n* = 0)	—	—
Ethnicity		
White British	17	73.9
White other	2	8.7
Black British	1	4.3
Hispanic	1	4.3
Mixed	1	4.3
Prefer not to say	1	4.34
Missing (*n* = 0)	—	—
DSM‐V eating disorder diagnosis		
AN‐R	15	65.2
AN‐BP	1	4.3
A‐AN	7	30.4
Missing (*n* = 0)	—	—
DSM‐5 comorbidity diagnosis^b^		
Major depressive disorder	7	30.44
Anxiety disorder (specific phobia, social anxiety, generalised anxiety)	11	47.83
Autism	1	4.35
Oppositional	3	13.04
Missing	5	21.74
At least 1 axis 1 disorder	13	56.52
Hospitalisation		
Y	0	0.00
N	23	100.00

^a^ Data presented for the eight young people who received MFT‐AN.

^b^ Diagnosis predicted with the Development and Wellbeing Assessment (DAWBA) (Goodman et al. 2000).

Abbreviations: %mBMI, percentage of median body mass index; A‐AN, other specified feeding and eating disorder (atypical anorexia nervosa); AN‐BP, anorexia nervosa (binge‐purge subtype); AN‐R, anorexia nervosa (restrictive subtype); DSM‐5, Diagnostic and Statistical Manual (fifth ed.).

### 3.1 Qualitative Findings

**FIGURE 1:**
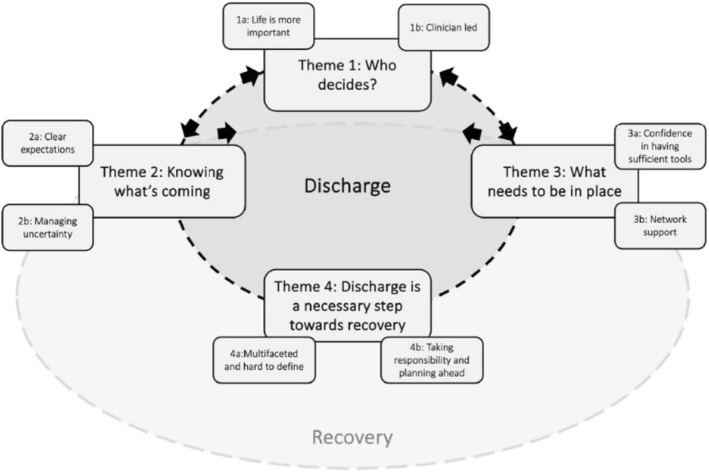
Proposed model of inter‐connected themes.

Four themes and eight subthemes were generated and are understood to be inter‐connected (see Figure 1). The data are inherently influenced by the analysing authors and their own experiences and biases.

### 3.2 Theme 1: Who Decides?

Participants spoke about the importance of collaborative decision‐making in the discharge process. They described a need to reach consensus between themselves, their caregivers and clinician(s) about when to end treatment. For themselves, young people said a good indicator to end was when they felt they had gotten the most out of sessions. They also spoke about the importance of their caregivers and clinician(s) feeling confident that the young person was able to manage without ongoing intervention. Who initiated discussions around discharge differed, however, most spoke about the importance of consensus.

I mean, you know, I was very sad, and I wanted to prolong it. But, yeah, I think I was happy when it ended when it did because it was a good time. Yeah, it was good because it wasn't, like, rushed or anything. It was when I really felt, okay, I can do it on my own.

(young person 17)

#### 3.2.1 Life Is More Important

Some young people said specifically that they felt ready to be discharged once the content of sessions had become exhausted or they had become bored; ‘when you're going to the sessions, and you don't really have that much to talk about because everything is good’ (young person 15). More generally, there was a broad sense between participants that a good sign that treatment could end was when they were running out of topics to cover or that attending sessions was getting in the way of doing other, more important things.

…feeling like, there was a point where I'd kind of be like, Ergh I have an appointment today, and I think at that point that's the time to leave, when it actually becomes more of a nuisance to go.

(young person 13)

Other young people described a shift in mindset, finding that life was more important than the illness or treatment. They said this was an important turning point in preparation for discharge. At discharge, one young person explained: ‘positives are much better than the negatives, and trying to think long term rather than short term’ (young person 19). As recovery progressed, young people's worlds widened, and they began to realise there was more to their life than the eating disorder.

At the time, I was very worried about being discharged, and I was like, Oh no, what happens now? But looking back, yes. Also because I was starting a new college in September and I have such long days. We start at eight and go to about five every day. So there was no way I was going to be like, Sorry guys. We just got to a miss a tap class so I can go and speak to my therapist.

(young person 12)

#### 3.2.2 Clinician Led

Several young people said it was their clinicians who initiated conversations about discharge. This was met with some anxiety, however, ultimately led to collaborative planning and preparation prior to discharge.

And [therapist] was sitting there going ‘no you are’ [ready]. You know, he [therapist] trusted me not to be weighing myself and having the meal plan. And to stop the treatment when I wasn’t sure. He just knew that wasn’t going to be happening… that was so powerful you know, I was still so worried and anxious.

(young person 4)

### 3.3 Theme 2: Knowing What's Coming

There was a strong sense from young people that having clear expectations regarding treatment length, likely outcomes and the possibility of ongoing symptoms after discharge was important to establish from the outset of treatment and to revisit as discharge approached.

Well, we, sort of, spaced out a lot less frequently to the point where when things were looking good, we went down to, like, once every four weeks and then once a month and then on, like, the second to last one, we decided that we're gonna have one more a month later, and then we're going to finish.

(young person 15)

#### 3.3.1 Expectations Set

Several young people shared that it was important for clinicians to be clear that some eating disorder cognitions are likely to persist beyond discharge and that this does not necessarily mean people need to remain in treatment. One young person noted that ‘they made it clear that I was in… a frame of mind that was good enough for me to be, sort of put on my own track’ (young person 3). The expectation was set that they were in a ‘good enough’ place for discharge, and there was recognition that there would be continued progress in terms of recovery post‐discharge.

We spoke about it with [therapist] and she was saying that you don’t go away from it being 100, like, fully recovered because then they wouldn’t be doing their job because then you’ve got to do some on your own….

(young person 5)

#### 3.3.2 Managing Uncertainty

Anxiety around discharge was mentioned specifically by three young people. To manage this, young people spoke about how useful it was to discuss this openly during sessions and for their clinician to remind them of the tools they had developed, how much progress had been made and that discharge was an important step towards recovery. One young person acknowledged ‘there's always going to be that initial sense of caution, anxiety in terms of, wow, I'm going to be doing this on my own now’ (young person 4). Others felt anxious about the loss of structure of sessions, but reassurance from their therapists towards the end was containing.

I was a bit scared of not having that sort of stability of it. But I think by the end of the session, I was like, okay I feel good about it now. And I think it was just because I was able to kind of calm myself down. And I kind of realised that I actually have all of these sort of strategies to help my… help myself, sort of thing.

(young person 10)

### 3.4 Theme 3: Things That Need to Be in Place

Along with a change in mindset, young people spoke about needing to have the right tools and scaffolding (e.g. support network) in place to continue to work towards recovery post‐discharge. Young people described having learnt to manage their illness, through working alongside their family, friends and others in their support network.

… put all of, like, the new stuff that I knew, like, just actually like, put that into action and just start like reworking like the way I live my life pretty much.

(young person 1)

#### 3.4.1 Confidence in Having Sufficient Tools

One important shift that was identified by six young people was the need to have confidence in the tools they gained throughout FT‐AN. One young person explained, ‘I think the right time to end the treatment is when the emotional side of it is healed and the eating disorder no longer controls you. So, as I said, you have those thoughts, but, you know, you're able to ignore them, and they're occasional’ (young person 18). The ability to manage and challenge eating disorder cognitions was described as very important.

I think it was the right time because it was almost as if they had given us the tools to be able to deal with things. And there wasn’t much else they could really do for me because it was kind of in my own hands now.

(young person 8)

#### 3.4.2 Network Support

Having a strong support network in place was described as an integral part of being ready for discharge. Young people described how this often builds throughout treatment, however, it was stated as an important thing to specifically focus on in preparation for discharge. Six young people specifically identified family and social networks as key to address.

Obviously, there's still going to be the support from my family, and I know that they're always there if I need them.

(young person 8)

### 3.5 Theme 4: Discharge Is a Necessary Step Towards Recovery

At the point of discharge, there were different experiences of how far along the recovery journey participants self‐identified. Nine young people said they were recovered at discharge, 11 said they were partly, three said they were not recovered. Four of those who did not self‐identify as recovered at dischare added that they recovered after discharge. However far along young people felt they were in this journey, all recognised that the recovery process continued after discharge and that discharge aided this process.

I think things have actually gotten better since I've left as well, because it's forced me to—forced, but in a good way. It's forced me to be more independent, and I think now, recovery is a choice.

(young person 11)

#### 3.5.1 Multifaceted and Hard to Define

Young people had differing definitions of recovery and almost all said it was difficult to define with absolute certainty. One young person shared: ‘the whole recovered, I don't think it's as simple as you know, black and white’ (young person 4). Another said the concept of recovery was confusing: ‘before I'd be like, oh, I have an eating disorder. But now I'm like, do I say I have one? Or am I like, I had an eating disorder? I find that very hard to differentiate’ (young person 12).

I don’t know. I mean people have different definitions of what it means to be recovered. You know, for some people, it’s the physical. For some people, it’s the mental, you know, you could say recovered is knowing that’s never gonna happen again or, be able to show, teach other people about your experience.

(young person 4)

There was a sense for many young people that there are multiple aspects to recovery, and that it isn't just the physical aspect of weight gain. Many described the importance of also considering broader aspects of mental, emotional and social well‐being. One young person explained that following discharge, ‘when you've got to do some on your own, and that's like when you can do it fully on your own, that's when you feel more like you're fully recovered’ (young person 5). Discharge appeared to facilitate recovery in terms of life getting bigger, and the eating disorder becoming less of a focus. Instead, other aspects of life, such as friendships, family and school became more important.

It was kind of seeing my life expand again and not being stuck in this hole of food and exercise. And that was all I thought about. I actually have friends now, I can focus on school now, and I can make spontaneous plans. It just feels like my life is just brighter again.

(young person 13)

#### 3.5.2 Taking Responsibility and Planning Ahead

Young people said it was important to plan ahead and to continuously set goals and use skills post‐discharge. One young person spoke about how important discharge was in facilitating them to fully take responsibility for their recovery.

It was more like taking that responsibility on myself. Actually putting things into a practice that I had learned. So it was definitely an ongoing process. So, yeah, outside of the labels of recovered, not recovered. It was just the process of recovery, you know, didn't stop when I left treatment.

(young person 4)

Many acknowledged the possibility of relapse in the future, with one young person sharing: ‘if there's, like, really big, stressful events in my life or, there's like a change, or there's just something where I feel like out of control and the thoughts just kind of come back … as the time passes, I think the chance gets smaller and smaller. And I don't think that means everyone needs to stop being vigilant and stop like watching out for signs and stuff like that and myself included, and I'm very aware of signs’ (young person 18). There was a sense that, with the right tools and support, and whilst continuing to implement learning, life continues to grow.

And I do think with the right tools and the right system, there’s just a lot of factors that will determine whether someone can recover. And I think family is a huge factor in that, and I think it’s acknowledging all the factors and tackling them that will ensure the best success of treatment.

(young person 18)

## 4 Discussion

The aim of this study was to explore young people's perspectives on the timing of discharge from FT‐AN and how this relates to their perception of recovery. Four main themes were generated. Three described the ‘right time’ for discharge, and one related more to defining recovery. All themes were inter‐related and influenced one another. Young people recognised that once they were discharged from treatment, they were then able to make the final steps towards recovery. With hindsight, many reflected that if they had remained in treatment longer this may have hindered their progress.

As young people prepared for discharge, they reflected upon who should determine the timing. Some participants found they reached a natural conclusion to FT‐AN when sessions became less engaging (even ‘boring’) or when other life priorities took over. Others felt this discussion should be initiated by clinicians. Nevertheless, most said clinicians and parents needed to be part of guiding this decision together with the young person. This fits with previous research, which highlights the importance of therapists and young people collaborating on decisions about ending (Lindstedt et al. 2015). This emphasis on joint decision‐making also aligns with existing literature that recognises the importance of engagement, collaborative formulation, and clear treatment planning in FT‐AN (Baudinet et al. 2024; James et al. 2025; Baudinet et al. 2021a, 2021b).

Young people also emphasised in the current study the value of knowing what was coming and feeling like they had some tools in place to feel ready for discharge. They benefitted from having clear expectations about session frequency, treatment length, as well as expected outcomes at discharge, which were communicated from the outset of treatment and revisited as discharge approached. This does not necessarily mean an explicit number of sessions, rather expectations about duration of treatment, frequency of sessions and potential anxiety about ending. Several said conversations about discharge were associated with increased anxiety and that they needed continued discussion and encouragement before they felt confident to end. This fits with a key theoretical concept within the FT‐AN model that treatment needs to support families to reach a place of ‘safe uncertainty’ (Mason 1993). Initially, ‘safe certainty’ is provided by the FT‐AN structure (frequent sessions, parental support at meals, etc.) and knowledge of the multi‐disciplinary team. Then, across treatment, more flexibility and ‘safe uncertainty’ is introduced to allow young people to resume independence around eating, take safe risks in a developmentally appropriate way, and take responsibility for the recovery process. The current findings suggest discharge is an important step in truly handing over responsibility for recovery to the young person with family support.

Previous studies have highlighted the importance of intentionally building confidence and self‐efficacy in parents and young people, in order to facilitate ongoing recovery‐oriented change (Konstantellou et al. 2022; Socholotiuk and Young 2022). For adults with eating disorders, improving tolerance of uncertainty (particularly early in treatment) has also been associated with improved symptoms at discharge (Reilly et al. 2021). By intentionally reducing session frequency in the middle and later phases of treatment, clinicians are setting a clear expectation that the young person (with family support) is expected to take more responsibility in the later phases of treatment. Addressing fears around lapses and relapse was described as important in helping the young person and family take ownership of the recovery process and feel prepared for discharge. It also signalled to the young people that the team felt confident they could manage without ongoing support.

In previous studies, young people have commented on how detrimental it can be for clinicians and teams to over‐focus on eating behaviours at the expense of identity formation at the end of treatment (Conti et al. 2021). Identity functioning has been associated with illness stage in AN (Croce et al. 2024), and enhancing a sense of achievement across various life domains (e.g., academic, hobbies, etc.) can promote broader adolescent identity development during recovery (Nilsen et al. 2020). Treatment can provide an improved sense of self and self‐confidence for adolescents (Wallis et al. 2017). Research suggests that improvements in motivation to change often occur alongside a connection to values and identity formation (Rankin et al. 2023). Young people in the current study emphasised the importance of not becoming perfectionistic about recovery; as young person 4 said ‘I don't think it's as simple as, you know, black and white.’ Instead, prioritise discharge when things are ‘good enough’.

The current study also highlights the importance of discharge as a step towards recovery, as opposed to something that happens when someone is recovered. Young people spoke about how discharge enabled them to feel truly independent and that letting go of the ‘safety net’ of treatment allowed for the next step in this process. In a way, discharge seems to aid in ongoing adolescent identity development and a move away from the illness.

Unsurprisingly, young people in the current study struggled to define ‘recovery’, something that has been shared in the broader literature for some time (Bardone‐Cone et al. 2018; Baudinet and Eisler 2024a; Dawson et al. 2015; Kerimler et al. 2024). In this study, young people talked about recovery as an evolving, multifaceted process that continued beyond discharge. They said both physical and psychosocial change is important to consider, with physical recovery typically preceding cognitive and emotional recovery. Many young people spoke about how recovery might involve experiencing some ongoing eating disorder cognitions and urges, yet choosing not to respond to them. Given this lack of clarity on how to define recovery, basing the timing of discharge on reaching ‘recovery’ is problematic. The current findings suggest that discharge is appropriate when there is consensus between the young person, their family members, and clinician(s) that sufficient tools and support are in place to manage without ongoing sessions.

Young people in the current study expected there to be future ‘bumps in the road’ (e.g. life transitions and changes, personal challenges, etc.) that could trigger eating disorder cognitions and behaviours. Data suggest there are genetic and neuro‐temperamental correlates to AN (Frank et al. 2019; Kaye et al. 2013; Watson et al. 2019) meaning some illness risk and maintenance factors may persist. Seven‐year follow‐up data from the same recruiting service suggest that most do not continue having an eating disorder, but many will experience other mental health difficulties, such as anxiety and depression (Simic et al. 2022; Stewart et al. 2022). Recent novel treatment developments have demonstrated that targetting social connectedness may be useful in supporting broader social functioning and recovery for people with restrictive eating disorders (Baudinet, Stewart, et al. 2021; Baudinet et al. 2022). Future research is needed on the interaction between eating disorder recovery, broader social functioning and other co‐occurring mental health difficulties.

Furthermore, whilst eating disorder focused family therapy models utilise a phased approach to treatment, with specific therapeutic aims characterising each phase, research is yet to look specifically at the impact of completing each phase and treatment outcome. It may be pertinent to look at potential mechanisms of change specific to FT‐AN (e.g., parental confidence and self‐efficacy, improvements in parent‐child communication), and consider their temporal sequence alongside other well‐recognized mechanisms of change (such as early weight gain).

## 5 Strengths and Limitations

One of the main strengths of this study is the inclusion of young people's voices alone. More common in the literature is the voice of parents/caregivers and professionals combined with that of the young people. The sample size is also a strength of this study and allows for greater confidence and potential generalisability of the findings.

Nevertheless, this study has several limitations. Firstly, the themes and conclusions were not checked by the participants themselves, reducing the potential validity of the conclusions drawn. Young people were also interviewed at different points post‐discharge, with some having completed treatment just a few weeks prior to completing the interview, while others having been out of treatment for nearly two years. To increase specificity of the findings, it would be useful to see how young people's understanding of discharge and recovery may shift in the years post‐discharge. Also, the sample of young people who agreed to participate in the study may represent a cohort with a positive experience of treatment and recovery; indeed all had largely restored both physical and psychological health through their treatment. Notably, however, over 50% of the sample had comorbid mental health concerns, which aligns with previous audit data from our service (Simic et al. 2022; Stewart et al. 2022). Future research could extend to treatment non‐completers, or those not responding to treatment, to determine whether discharge processes should be adapted in these circumstances. The sample were predominantly white females and recruited from one service in southeast London. Treatment was mostly face‐to‐face, meaning the experiences reported may not be generalisable to other formats. Recent data suggests online is valued but face‐to‐face is preferred by most, particularly in the early phases of treatment (Baudinet, Konstantellou, et al. 2023). Whether the process around discharge needs different thinking for solely online FT‐AN is yet to be examined. Finally, whilst this paper offered the unique opportunity to hear from young people, parent and caregiver views were absent. Parents and careregivers may hold different views on the ‘right’ time for discharge and meaning of recovery. As they are a key resource in treatment, future research could compare and contrast their perspectives with data from the current study to promote a shared understanding about how to end treatment well.

## 6 Conclusions

The current study highlights the unique and varied needs young people have at both the specific point of discharge and as they continue towards recovery after treatment. The current findings demonstrate that ending treatment is not about attending a specific number of sessions or reaching a certain weight. Rather, it is about supporting young people to feel confident in their ability to manage without treatment. This is somewhat at odds with clinical research trials, in which a set number of sessions is typically prescribed. In real‐world clinical settings, addressing the multifaceted needs of each individual and their family, whilst also looking forward and communicating realistic expectations about discharge and recovery from the beginning of treatment, appears key. It seems that ongoing commitment to change post‐discharge is paramount, and encouraging young people and families to take ‘safe risks’ seems to be an important part of ending and taking ownership for recovery. Some may see ongoing cognitions as signalling the need for more treatment, however, current findings suggest staying in treatment may inhibit the final stages of recovery. Holding young people in treatment longer than necessary may inadvertently signal that change is too risky and that the multi‐disciplinary team do not think they are able to cope without ongoing support.

## Ethics Statement

The Stanmore Research Ethics Committee London (IRAS: 234354; REC: 20/LO/0839) provided ethical approval for this study.

## Consent

All participants provided written informed consent or assent.

## Conflicts of Interest

U.S. receives salary support from the NIHR Mental Health Biomedical Research Centre (BRC) at the South London and Maudsley NHS Foundation Trust and King's College London (KCL). She is also supported by the Medical Research Council/Arts and Humanities Research Council/Economic and Social Research Council Adolescence, Mental Health and the Developing Mind initiative as part of the EDIFY programme (Grant MR/W002418/1). J.B., I.E. and M.S. receive royalties from Routledge for a published treatment manual for multi‐family therapy for anorexia nervosa.

## Data Availability

Data are made available upon reasonable request.
